# Instilação Intrapericárdica de Cisplatina para Derrame Pericárdico Maligno: Uma Experiência em um Único Centro

**DOI:** 10.36660/abc.20220912

**Published:** 2023-10-20

**Authors:** Paulo Medeiros, Jorge Rodrigues, António Gaspar

**Affiliations:** 1 Hospital de Braga Braga Portugal Hospital de Braga , Braga – Portugal

**Keywords:** Derrame Pericárdio, Instilação de Medicamentos, Pericardiocentese, Antineoplásicos Alquilantes

## Introdução

O envolvimento do coração e do pericárdio ocorre em cerca de 10% de todos os pacientes com câncer e tem impacto significativo na sobrevida. ^1^ Carcinomas de pulmão e mama, melanoma e linfoma são os tumores malignos mais comuns que afetam o coração e o pericárdio. ^2^ O envolvimento do coração é um marcador de prognóstico muito ruim, representando doença neoplásica em estágio terminal.

A descompressão pericárdica via pericardiocentese resulta no alívio imediato da dispneia e desempenha um papel importante no tratamento sintomático. ^3^ Contudo, não é incomum a necessidade de múltiplos procedimentos no mesmo paciente. Nas últimas décadas surgiram relatos sobre a utilização de agentes citotóxicos para pericardiodese química, mas a literatura é escassa e nenhum documento de sistematização foi publicado. Os autores tiveram como objetivo avaliar a recorrência de derrame pericárdico maligno clinicamente significativo em pacientes submetidos à pericardiocentese e instilação de cisplatina.

## Métodos

Este foi um estudo observacional transversal retrospectivo. Este estudo está em conformidade com a Declaração de Helsinque e foi conduzido seguindo os requisitos do comitê de ética local.

Seleção de pacientes: Os autores coletaram dados de pacientes com idade ≥18 anos, internados em seu hospital por derrame pericárdico maligno entre janeiro de 2019 e janeiro de 2022, que foram submetidos à pericardiodese química com cisplatina. O único critério de exclusão foi a dependência total do paciente (status 4 de desempenho do Eastern Cooperative Oncology Group).

Dados clínicos e definições: Derrame pericárdico significativo foi definido por critérios ecocardiográficos: espessura do espaço pericárdico de pelo menos 20mm, alterações respiratórias exageradas nas velocidades E mitral e tricúspide, veia cava inferior >20 mm e variação <50% com a respiração e sinais de comprometimento do enchimento do ventrículo direito. A combinação de hipotensão e critérios ecocardiográficos definiu tamponamento cardíaco. A recidiva foi definida como internação por derrame pericárdico significativo, com ou sem tamponamento.

Técnica de instilação: A pericardiocentese foi realizada por via subxifoide ou apical. O espaço pericárdico foi deixado em drenagem livre até que menos de 50 mL/24h de líquido fossem coletados. Duas ou três instilações da preparação citotóxica (10 mg de cisplatina diluída em 20 mL de soro fisiológico) foram administradas por paciente em intervalos de 24 horas, com reavaliação ecocardiográfica antes de cada instilação. Após todas as instilações, o cateter pericárdico foi retirado.

## Resultados

Onze pacientes foram tratados com cisplatina intrapericárdica. As características basais estão descritas na Tabela 1 . A média de idade foi de 57 ± 14 anos (mínimo 36 anos, máximo 82 anos) e o sexo feminino foi predominante (64%; n=7).

**Tabela 1:** – Características basais e diagnóstico de câncer

Paciente nº.	Sexo	Anos de idade)	Diagnóstico de câncer	Diagnóstico conhecido antes da admissão
1	F	36	Adenocarcinoma de mama	Sim
2	M	40	Adenocarcinoma pulmonar	Sim
3	F	52	Adenocarcinoma pulmonar	Sim
4	F	52	Adenocarcinoma pulmonar	Sim
5	M	54	Adenocarcinoma de células renais	Não
6	M	57	Adenocarcinoma pulmonar	Sim
7	F	61	Adenocarcinoma pulmonar	Sim
8	F	63	Adenocarcinoma pulmonar	Sim
9	F	68	Adenocarcinoma pulmonar	Sim
10	F	73	Adenocarcinoma gástrico	Sim
11	M	82	Adenocarcinoma de próstata	Não

### Derrame pericárdico e instilação de cisplatina

A maioria dos pacientes apresentou tamponamento cardíaco; o volume médio de drenagem foi de 800±500 mL. Esses pacientes foram submetidos a duas instilações de cisplatina, exceto nos casos com história prévia de derrame pericárdico, nos quais foram realizadas três instilações. Em relação aos efeitos colaterais, três pacientes apresentaram fibrilação atrial de novo e dois tiveram febre autolimitada. Nenhum paciente apresentou complicações graves relacionadas à técnica de pericardiocentese ou à presença do cateter pericárdico. Os detalhes individuais são relatados na Tabela 2 .

**Tabela 2:** – Características do derrame pericárdico e da instilação de cisplatina

Paciente nº.	Tamponamento na admissão	Volume drenado (mL)	Aparência macroscópica	Nº de instilações de cisplatina	Efeitos colaterais	Recorrência de derrame durante o acompanhamento	Tempo desde a instilação até a morte (dias)
1	Sim	500	Serohemático	2	FA	Não	7
2	Sim	1700	Serohemático	2	-	Não	87
3	Não	800	Citrino	3	-	Não	233
4	Sim	1120	Hemorrágico	2	-	Não	528
5	Não	850	Hemorrágico	2	-	Não	148
6	Sim	1900	Hemorrágico	2	Febre	Não	438
7	Sim	850	Hemorrágico	2	FA	Não	-*
8	Sim	800	Hemorrágico	2	FA	Não	386
9	Não	1000	Hemorrágico	3	Febre	Sim	284
10	Sim	1700	Hemorrágico	2	-	Não	235
11	Não	400	Hemorrágico	2	-	Não	134

FA: fibrilação atrial. *Paciente vivo durante o acompanhamento.

### Acompanhamento

O seguimento médio foi de 290 dias. Durante o acompanhamento, dez pacientes morreram em média de 248 dias por pericardiodese química. Nenhum dos pacientes morreu de causas cardiovasculares. A recorrência de derrame pericárdico ocorreu em um caso de adenocarcinoma pulmonar após aproximadamente 12 meses da instilação de cisplatina.

## Discussão

Os autores descrevem uma experiência unicêntrica de pericardiodese química com cisplatina após pericardiocentese percutânea, em pacientes com derrame pericárdico maligno.

Um dos primeiros relatos humanos de tentativa de controle de derrame pericárdico recorrente com instilação intrapericárdica de uma substância foi publicado em 1953 por Bachman KP et al., utilizando ouro radioativo. ^4^ Desde então, outros compostos foram testados, como a tetraciclina, ^5^ bleomicina, ^6^ colchicina, ^7^ entre outros. Agentes intrapericárdicos também foram testados em outros contextos, como na síndrome de Dressler ^8^ e pericardite aguda após estudos eletrofisiológicos. ^9^

A maior série de pacientes tratados com cisplatina intrapericárdica foi publicada por Maisch et al., ^10^ e Tomkowski et al., ^11^ incluindo 42 e 46 pacientes, respectivamente. Assim como no presente estudo, a maioria dos pacientes apresentava câncer de pulmão. Em Maish et al., ^10^ uma única instilação intrapericárdica de cisplatina evitou a recorrência de derrame pericárdico hemodinamicamente relevante durante os primeiros três meses de acompanhamento em 92,8% dos pacientes. Embora o prognóstico geral tenha sido ruim, nenhum dos pacientes morreu devido a tamponamento cardíaco. Em Tomkowski et al., ^11^ nenhum acúmulo de grandes quantidades de líquido pericárdico foi alcançado em 93,5% dos pacientes após a instilação de cisplatina. Todos os pacientes inscritos morreram devido a malignidade avançada. A fibrilação atrial transitória foi o efeito colateral mais comum. Outros efeitos colaterais relatados incluem dor, febre, náusea e taquicardia ventricular não sustentada. ^12 , 13^

Esta série relata achados semelhantes, incluindo um controle bem-sucedido do acúmulo de líquido pericárdico. Esses resultados apoiam a ideia de uma opção segura e eficaz para pacientes com derrame pericárdico maligno, que provavelmente apresentarão recorrências após uma pericardiocentese bem-sucedida. A sobrevida global é baixa. A pericardiodese química visa aliviar os sintomas e pode até prevenir algumas mortes por tamponamento cardíaco; não obstante, não há evidência de benefício de sobrevivência, uma vez que os pacientes ainda apresentam progressão da doença neoplásica. Tanto quanto é do conhecimento dos autores, esta é a primeira série publicada de instilação intrapericárdica de um agente esclerosante/quimioterápico desenvolvido em Portugal.

Este estudo tem algumas limitações importantes. Primeiro, este é um estudo retrospectivo e observacional realizado em uma pequena amostra de pacientes. Segundo, por razões éticas, não houve grupo de controle; como tal, não pudemos avaliar o efeito exclusivo da cisplatina independentemente do tratamento padrão da neoplasia primária. Além disso, não foi possível realizar ecocardiogramas de acompanhamento programados para avaliar a reacumulação subclínica de líquido pericárdico. Estes resultados merecem uma investigação mais aprofundada, nomeadamente em estudos prospectivos e maiores.

## Conclusões

A instilação intrapericárdica de cisplatina parece ser uma opção de tratamento eficaz e segura para pacientes com derrame pericárdico maligno. Embora não haja evidência de aumento da sobrevida, estes dados sugerem que o alívio dos sintomas e a prevenção da recorrência são provavelmente alcançáveis. Portanto, a pericardiodese deve ser incluída no arsenal dos profissionais de cuidados paliativos.
