# Supplementary material for: Instilação Intrapericárdica de Cisplatina para Derrame Pericárdico Maligno: Uma Experiência em um Único Centro
Source: Arq Bras Cardiol. 2023 Oct 20;120(10):e20220912. [Article in Portuguese] doi: 10.36660/abc.20220912 (PMC10593389; doi:10.36660/abc.20220912)
Supplement: Translation

# Translation: Intrapericardial Cisplatin Instillation for Malignant Pericardial Effusion: A Single-center Experience

**Authors:** Paulo Medeiros, Jorge Rodrigues, António Gaspar

**Affiliations:** 1 Portugal Hospital de Braga, Braga – Portugal

**Keywords:** Pericardial Effusion, Instillation, Drugs, Pericardiocentesis, Antineoplastic, Agents, Alkylating

## Introduction

Heart and pericardium involvement occurs in about 10% of cancer patients, significantly impacting survival. ^1^ Lung and breast carcinomas, melanoma, and lymphoma are the most common malignant tumors affecting the heart and pericardium. ^2^ Involvement of the heart is a marker of very poor prognosis, representing end-stage neoplastic disease.

Pericardial decompression via pericardiocentesis results in immediate dyspnea relief and plays an important role in symptomatic treatment. ^3^ Notwithstanding, the need for multiple procedures in the same patient is not uncommon. During the last decades, reports emerged regarding the utilization of cytotoxic agents for chemical pericardiodesis, but literature is scarce overall, and no systematization document has been published. The authors aimed to assess the recurrence of clinically significant malignant pericardial effusion in patients submitted to pericardiocentesis and cisplatin instillation.

## Methods

This was a retrospective cross-sectional observational study. This study complies with the Declaration of Helsinki and was conducted following the local ethics committee requirements.

Patient selection: The authors collected data from patients aged ≥18 years, admitted to their hospital due to malignant pericardial effusion between January 2019 and January 2022, who were submitted to chemical pericardiodesis with cisplatin. The only exclusion criterion was total patient dependency (Eastern Cooperative Oncology Group Performance Status of 4).

Clinical data and definitions: Significant pericardial effusion was defined by echocardiographic criteria: pericardial space thickness of at least 20mm, exaggerated respiratory changes in the mitral and tricuspid E velocities, inferior vena cava >20 mm and <50% variation with respiration and signs of right ventricle filling impairment. The combination of hypotension and echocardiographic criteria defined cardiac tamponade. Recurrence was defined as a hospitalization due to significant pericardial effusion, with or without tamponade.

Instillation technique: Pericardiocentesis was performed using a subxiphoid or apical approach. Pericardial space was left on free drainage until less than 50 mL/24h of fluid was collected. Two or three instillations of the cytotoxic preparation (10 mg of cisplatin diluted in 20 mL of normal saline) were administered per patient in 24h intervals, with echocardiography reassessment before each instillation. After all the instillations, the pericardial catheter was removed.

## Results

Eleven patients were treated with intrapericardial cisplatin. Baseline characteristics are described in Table 1 . The mean age was 57 ± 14 years (minimum 36 years, maximum 82 years), and female sex was predominant (64%; n=7).

**Table 1:** – Baseline characteristics and cancer diagnosis

Patient No.	Sex	Age (years)	Cancer diagnosis	Diagnosis known before admission
1	F	36	Breast adenocarcinoma	Yes
2	M	40	Lung adenocarcinoma	Yes
3	F	52	Lung adenocarcinoma	Yes
4	F	52	Lung adenocarcinoma	Yes
5	M	54	Renal cell adenocarcinoma	No
6	M	57	Lung adenocarcinoma	Yes
7	F	61	Lung adenocarcinoma	Yes
8	F	63	Lung adenocarcinoma	Yes
9	F	68	Lung adenocarcinoma	Yes
10	F	73	Gastric adenocarcinoma	Yes
11	M	82	Prostate adenocarcinoma	No

### Pericardial effusion and cisplatin instillation

Most patients presented with cardiac tamponade; the mean drainage volume was 800±500 mL. These patients were submitted to two cisplatin instillations, except in cases with previous history of pericardial effusion, in which three instillations were performed instead. Regarding side effects, three patients experienced *de novo* atrial fibrillation, and two had self-limited fever. No patients experienced serious complications related to the pericardiocentesis technique or the presence of the pericardial catheter. Individual details are reported in Table 2 .

**Table 2:** – Pericardial effusion and cisplatin instillation features

Patient No.	Tamponade at admission	Drained volume (mL)	Macroscopic appearance	No. of cisplatin instillations	Side effects	Effusion recurrence during follow-up	Time from instillation to death (days)
1	Yes	500	Serohematic	2	AF	No	7
2	Yes	1700	Serohematic	2	-	No	87
3	No	800	Citrine	3	-	No	233
4	Yes	1120	Hemorrhagic	2	-	No	528
5	No	850	Hemorrhagic	2	-	No	148
6	Yes	1900	Hemorrhagic	2	Fever	No	438
7	Yes	850	Hemorrhagic	2	AF	No	-*
8	Yes	800	Hemorrhagic	2	AF	No	386
9	No	1000	Hemorrhagic	3	Fever	Yes	284
10	Yes	1700	Hemorrhagic	2	-	No	235
11	No	400	Hemorrhagic	2	-	No	134

AF: atrial fibrillation. * Patient alive during follow-up.

### Follow-up

The mean follow-up was 290 days. During follow-up, ten patients died within a mean of 248 days from chemical pericardiodesis. None of the patients died from cardiovascular causes. Recurrence of pericardial effusion occurred in one case of lung adenocarcinoma after approximately 12 months from cisplatin instillation.

## Discussion

The authors describe a single-center experience on chemical pericardiodesis with cisplatin following percutaneous pericardiocentesis, in patients with malignant pericardial effusion.

One of the first human reports of an attempt to control recurrent pericardial effusion with intrapericardial instillation of a substance was published in 1953 by Bachman K.P. et al., using radioactive gold. ^4^ Since then, other compounds have been tested, such as tetracycline, ^5^ bleomycin, ^6^ colchicine, ^7^ among others. Intrapericardial agents have also been tested in other settings, such as in Dressler’s syndrome ^8^ and acute pericarditis following electrophysiology studies. ^9^

The largest series of patients treated with intrapericardial cisplatin were published by Maisch et al. ^10^ and Tomkowski et al., ^11^ including 42 and 46 patients, respectively. As in the present study, most patients had lung cancer. In Maish et al., a single intrapericardial cisplatin instillation prevented the recurrence of a hemodynamically relevant pericardial effusion during the first three months of follow-up in 92.8% of the patients. Although the overall prognosis was poor, none of the patients died due to cardiac tamponade. In Tomkowski et al., no accumulation of large amounts of pericardial fluid was achieved in 93.5% of patients after cisplatin instillation. All enrolled patients died because of advanced malignancy. Transient atrial fibrillation was the most common side effect. Other reported side effects include pain, fever, nausea, and non-sustained ventricular tachycardia. ^12 , 13^

This series reports similar findings, including a successful control of pericardial fluid accumulation. These results support the idea of a safe and effective option for patients with malignant pericardial effusion, who are likely to experience recurrences after successful pericardiocentesis. The overall survival is poor. Chemical pericardiodesis aims to alleviate symptoms and may even prevent some deaths due to cardiac tamponade; notwithstanding, there is no evidence of survival benefit, as patients still experience neoplastic disease progression. To the authors’ knowledge, this is the first published series of intrapericardial instillation of a sclerosing/chemotherapeutic agent developed in Portugal.

This study has some important limitations. First, this is a retrospective and observational study performed on a small sample of patients. Second, for ethical reasons, there was no control group; as such, we could not evaluate the sole effect of cisplatin independently of the standard treatment of the primary neoplasm. Also, scheduled follow-up echocardiograms to assess subclinical reaccumulation of pericardial fluid could not be performed. These results warrant further investigation, namely in prospective and larger studies.

## Conclusions

Intrapericardial instillation of cisplatin seems to be an effective and safe treatment option for patients with malignant pericardial effusion. Although there is no evidence of increased survival, this data suggests that symptom relief and recurrence prevention are likely achievable. Therefore, pericardiodesis should be included in the armamentarium of palliative care practitioners.
